# Assessment of the Physical and Emotional Health-Related Quality of Life Among Congestive Heart Failure Patients with Preserved and Reduced Ejection Fraction at a Quaternary Care Teaching Hospital in Coastal Karnataka in India

**DOI:** 10.3390/healthcare13151874

**Published:** 2025-07-31

**Authors:** Rajesh Kamath, Vineetha Poojary, Nishanth Shekar, Kanhai Lalani, Tarushree Bari, Prajwal Salins, Gwendolen Rodrigues, Devesh Teotia, Sanjay Kini

**Affiliations:** 1 Department of Healthcare and Hospital Management, Prasanna School of Public Health, Manipal Academy of Higher Education, Manipal 576104, India; vineetha.psphmpl2023@learner.manipal.edu (V.P.); gwendolen.psphmpl2023@learner.manipal.edu (G.R.); devesh.psphmpl2023@learner.manipal.edu (D.T.); 2 Department of Pediatric Oncology, Kasturba Medical College, Manipal, Manipal Academy of Higher Education, Manipal 576104, India; nishanth.kmcmpl2024@learner.manipal.edu; 3 Department of Cardiology, Kasturba Medical College, Manipal, Manipal Academy of Higher Education, Manipal 576104, India; 4 Directorate of Online Education, Manipal Academy of Higher Education, Manipal 576104, India; tarushree.bari@manipal.edu; 5 Department of Health Information Management, Manipal College of Health Professions, Manipal Academy of Higher Education, Manipal 576104, India; prajwal.salins@manipal.edu; 6 Department of Community Medicine, Kasturba Medical College, Manipal, Manipal Academy of Higher Education, Manipal 576104, India

**Keywords:** congestive heart failure (CHF), heart failure with preserved ejection fraction (HFpEF), heart failure with reduced ejection fraction (HFrEF), health-related quality of life (HRQoL), Minnesota living with heart failure questionnaire (MLHFQ), NYHA classification, chronic disease burden

## Abstract

**Introduction:** Congestive heart failure (CHF), a complex clinical syndrome characterized by the heart’s inability to pump blood effectively due to structural or functional impairments, is a growing public health concern, with profound implications for patients’ physical and emotional well-being. In India, the burden of CHF is rising due to aging demographics and increasing prevalence of lifestyle-related risk factors. Among the subtypes of CHF, heart failure with preserved ejection fraction (HFpEF), i.e., heart failure with left ventricular ejection fraction of ≥50% with evidence of spontaneous or provokable increased left ventricular filling pressure, and heart failure with reduced ejection fraction (HFrEF), i.e., heart failure with left ventricular ejection fraction of 40% or less and is accompanied by progressive left ventricular dilatation and adverse cardiac remodeling, may present differing impacts on health-related quality of life (HRQoL), i.e., an individual’s or a group’s perceived physical and mental health over time, yet comparative data remains limited. This study assesses HRQoL among CHF patients using the Minnesota Living with Heart Failure Questionnaire (MLHFQ), one of the most widely used health-related quality of life questionnaires for patients with heart failure based on physical and emotional dimensions and identifies sociodemographic and clinical variables influencing these outcomes. **Methods:** A cross-sectional analytical study was conducted among 233 CHF patients receiving inpatient and outpatient care at the Department of Cardiology at a quaternary care teaching hospital in coastal Karnataka in India. Participants were enrolled using convenience sampling. HRQoL was evaluated through the MLHFQ, while sociodemographic and clinical characteristics were recorded via a structured proforma. Statistical analyses included descriptive measures, independent *t*-test, Spearman’s correlation and stepwise multivariable linear regression to identify associations and predictors. **Results:** The mean HRQoL score was 56.5 ± 6.05, reflecting a moderate to high symptom burden. Patients with HFpEF reported significantly worse HRQoL (mean score: 61.4 ± 3.94) than those with HFrEF (52.9 ± 4.64; *p* < 0.001, Cohen’s d = 1.95). A significant positive correlation was observed between HRQoL scores and age (r = 0.428; *p* < 0.001), indicating that older individuals experienced a higher burden of symptoms. HRQoL also varied significantly across NYHA functional classes (χ^2^ = 69.9, *p* < 0.001, ε^2^ = 0.301) and employment groups (χ^2^ = 17.0, *p* < 0.001), with further differences noted by education level, gender and marital status (*p* < 0.05). Multivariable linear regression identified age (B = 0.311, *p* < 0.001) and gender (B = –4.591, *p* < 0.001) as significant predictors of poorer HRQoL. **Discussion:** The findings indicate that patients with HFpEF experience significantly poorer HRQoL than those with HFrEF. Older adults and female patients reported greater symptom burden, underscoring the importance of demographic-sensitive care approaches. These results highlight the need for routine integration of HRQoL assessment into clinical practice and the development of comprehensive, personalized interventions addressing both physical and emotional health dimensions, especially for vulnerable subgroups. **Conclusions:** CHF patients, especially those with HFpEF, face reduced HRQoL. Key factors include age, gender, education, employment, marital status, and NYHA class, underscoring the need for patient-centered care.

## 1. Introduction

Heart failure (HF) remains a significant global public health issue, affecting approximately 64.3 million individuals, or 0.8% of the global population [1,2]. It is a progressive condition characterized by the heart’s inability to pump sufficient blood to meet the body’s metabolic demands. In India, HF affects an estimated 1.3 to 4.6 million adults, with annual incidence rates ranging between 0.5 and 1.8 million [3]. Alarmingly, only 40% of patients survive beyond five years post-diagnosis, reflecting the severe impact of HF on patient outcomes [3]. The burden of HF is expected to rise, particularly in South Asian countries like India, due to shifting epidemiological patterns and increasing prevalence of risk factors such as hypertension and diabetes [4]. Despite therapeutic advancements, hospitalization and mortality rates remain high, largely due to the complex symptomatology and presence of comorbidities [4]. Clinical outcomes vary between patients: those with reduced ejection fraction (HFrEF) are generally at higher risk of cardiovascular mortality compared to those with preserved ejection fraction (HFpEF). In India, patients with HFrEF face a 37% readmission rate and a hospital mortality rate of 6.78%, emphasizing the seriousness of the condition [4,5].

Heart failure (HF) manifests through a distinct set of symptoms, including exertional dyspnea, lower limb edema, orthopnea and general manifestations such as chronic cough, fatigue, nausea, anorexia, chest discomfort and unintentional weight gain [2,4]. The severity and fluctuation of these symptoms, if inadequately managed, contribute significantly to reduced health-related quality of life (HRQoL) [6,7]. HRQoL encompasses a patient’s perception of how a disease affects their physical functioning, emotional state, social interactions and daily living [7]. In HF, persistent symptoms such as dyspnea and fatigue are strongly associated with frequent hospitalizations and diminished HRQoL [8]. Given that HF often represents the end stage of various cardiovascular conditions, evaluating HRQoL offers critical insights into both physical and emotional health, aiding clinicians in assessing treatment effectiveness [5].

HRQoL has emerged as a vital indicator in both clinical practice and research, serving to measure therapeutic outcomes and monitor disease progression [9]. Patients with HF often experience a progressive loss of physical independence and psychological distress due to restricted social participation, resulting in a significant decline in overall quality of life [10]. Despite medical advancements, HRQoL among individuals with chronic HF remains poor, particularly in low- and middle-income countries (LMICs), where the socio-economic impact is disproportionately high. LMICs bear a substantial share of the global cardiovascular disease (CVD) burden, accounting for nearly 80% of all CVD-related deaths. Between 2008 and 2017, high-income countries produced 81.1% of global CVD research while representing only 15% of the world’s population and contributed just 8.1% of CVD-related DALYs and 8.5% of deaths. In contrast, LMICs—home to 42% of the global population—contributed only 2.8% of CVD research but were responsible for 59.5% of DALYs and 57.1% of deaths due to CVD [11]. This imbalance highlights the urgent need for context-specific research and policies tailored to the unique challenges of HF in LMICs. Understanding and addressing HRQoL in HF patients provides a holistic measure of treatment success and informs the development of culturally and economically appropriate healthcare interventions [6].

Evidence from Western literature suggests that patients with heart failure (HF) experience declines in health-related quality of life (HRQoL) more frequently than mortality events [6]. Interestingly, several studies have found that younger HF patients report poorer HRQoL compared to older patients [4,12]. For example, Wong et al. (2013), through the CHARM study on mortality and morbidity reduction, categorized patients into five age groups and found that the youngest cohort (20–39 years) had the worst HRQoL with a mean MLHFQ score of 52.6, whereas those aged ≥70 years reported a significantly better score of 35.3 [12]. This disparity may be attributed to younger patients experiencing heightened psychological distress, including anxiety and depression, due to disrupted social, occupational and personal role expectations [13]. Reflecting this, current HF management guidelines underscore the importance of prioritizing quality of life (QoL) alongside clinical outcomes [6].

Multiple factors influence HRQoL in individuals with HF, including sociodemographic characteristics (such as age, sex and marital status), left ventricular ejection fraction (LVEF), disease duration, psychosocial well-being and comorbidities. Studies from countries including the United States, Canada, Brazil and across Europe consistently show that patients often value improvements in QoL more than prolonged survival [10]. Additionally, patients in advanced NYHA functional classes (III–IV) report significantly greater HRQoL impairment than those in lower classes (I–II) [4,12]. The 2022 guidelines jointly released by the Heart Failure Society of America (HFSA), American Heart Association (AHA) and American College of Cardiology (ACC) classify HF based on LVEF into reduced (≤40%), mildly reduced (40–49%), preserved (≥50%), and improved ejection fraction (HFimpEF) [14]. HFimpEF refers to patients initially diagnosed with reduced LVEF who later show a ≥10-point improvement and achieve LVEF > 40%, reflecting a positive therapeutic response. However, clinical comparisons across these categories remain limited [5].

Given these gaps, the present study seeks to assess and compare HRQoL among patients with congestive heart failure (CHF) with preserved and reduced ejection fractions, and to identify key sociodemographic factors influencing HRQoL at a tertiary care teaching hospital in coastal Karnataka. By exploring the physical and emotional domains of HRQoL and their determinants, the study aims to generate evidence that may support the development of patient-centered and stratified care strategies for individuals living with HF.

## 2. Methodology

### 2.1. Study Area and Design

A prospective, cross-sectional analytical study was conducted among patients diagnosed with CHF at the inpatient and outpatient cardiology services of a quaternary care teaching hospital in coastal Karnataka.

### 2.2. Participants and Sample Size

Patients aged 18 years and above with a confirmed CHF diagnosis were included, provided they could comprehend the study language and gave written informed consent. Those with cognitive or communication impairments or under 18 years of age were excluded. Participants were classified into HFrEF HFpEF groups. A convenience sampling method was used for recruitment, whereby eligible participants were enrolled consecutively as they presented at the study site during the data collection period. This approach was chosen due to time constraints, ease of access, and the availability of patients within the clinical setting. Based on G*Power v.3.1.9.7 analysis with a medium effect size (Cohen’s d = 0.5), 95% power and a 5% significance level, the estimated sample size was 210. Accounting for a 0.9% dropout rate, the final sample comprised 233 participants.

### 2.3. Tools Used

HRQoL was assessed using the validated Minnesota Living with Heart Failure Questionnaire (MLHFQ), comprising 21 items scored on a 6-point Likert scale. Additional data, including age, gender, education, employment, NYHA class, illness duration and latest LVEF values, were collected using a structured demographic and clinical data sheet. Based on 2022 AHA/ACC/HFSA guidelines, participants were categorized into HFrEF (LVEF ≤ 40%) and HfpEF (LVEF ≥ 50%).

### 2.4. Data Analysis and Statistical Methods

Data were entered into Microsoft Excel and analyzed using Jamovi v.2.6.23. Descriptive statistics summarized demographic and clinical profiles. Inferential tests, including an independent *t*-test and Spearman’s correlation, were used to assess group differences and variable associations. Multiple linear regression identified predictors of HRQoL.

### 2.5. Study Procedure and Ethical Considerations

Ethical clearance was obtained from the Institutional Ethics Committee (IEC). Participants were enrolled following eligibility screening and data collection was conducted through 20–25-min structured interviews in English or Kannada. Confidentiality and voluntary participation were ensured throughout the study.

## 3. Results

### 3.1. Sociodemographic and Clinical Profile

A total of 233 patients were enrolled in the study. The mean age was 59.6 years (SD = 9.37; range 33–82), and the mean MLHFQ score was 56.5 (SD = 6.05; range 42–70), indicating high symptom burden in the sample. Table 1 presents a comparative summary of the demographic and clinical characteristics of patients with HFrEF and those with HFpEF. Of the total sample (N = 233), 136 patients (58.4%) were diagnosed with HFrEF, while 97 patients (41.6%) had HFpEF. The majority of patients in both groups were within the age range of 46–65 years. Specifically, 21.5% of HFrEF and 18.9% of HFpEF patients were aged 56–65 years, while 15.5% of HFrEF and 10.3% of HFpEF patients were aged 46–55. Very few participants were under 45 years of age or over 75 years of age, with no HFpEF patients below age 46. Males predominated in the HFrEF group (51.9%) compared to females (6.4%). In contrast, the HFpEF group had a higher proportion of females (22.7%) than males (18.9%). In the HFrEF group, 26.2% were employed and 16.7% unemployed, while in the HFpEF group, 17.2% were employed and 12.4% unemployed. Retired patients constituted 15.5% and 12% of the HFrEF and HFpEF groups, respectively. Primary school education was the most common educational attainment in both groups (23.6% in HFrEF and 17.6% in HFpEF), followed by high school education (19.7% and 15.5% respectively). A smaller proportion had higher education (9.4% HFrEF; 4.3% HFpEF), while a few had no formal education. Most participants in both groups fell into NYHA Class III (31.3% HFrEF; 27.5% HFpEF), indicating moderate limitation of physical activity. HFrEF patients had a greater representation in Class IV (9.4%) compared to HFpEF patients (3%), while Class II accounted for 17.6% (HFrEF) and 11.2% (HFpEF). No patients were in Class I. The majority of patients reported living with heart failure for 2 to 3 years. In the HFrEF group, 17.6% had the condition for 2 years and 15.5% for 3 years. In the HFpEF group, 14.6% and 11.6% reported durations of 2 and 3 years, respectively. Notably, no HFpEF patients had a disease duration of 6 years, compared to 2.1% in the HFrEF group. A majority of participants in both groups were married, with 52.8% of HFrEF and 34.3% of HFpEF patients reporting married status. Widowed individuals were more common in the HFpEF group (7.3%) compared to HFrEF (4.3%). Only a small percentage of HFrEF patients (1.3%) were unmarried, with none in the HFpEF group.

### 3.2. Item-Level Analysis of MLHFQ Responses

**Figure 1:**
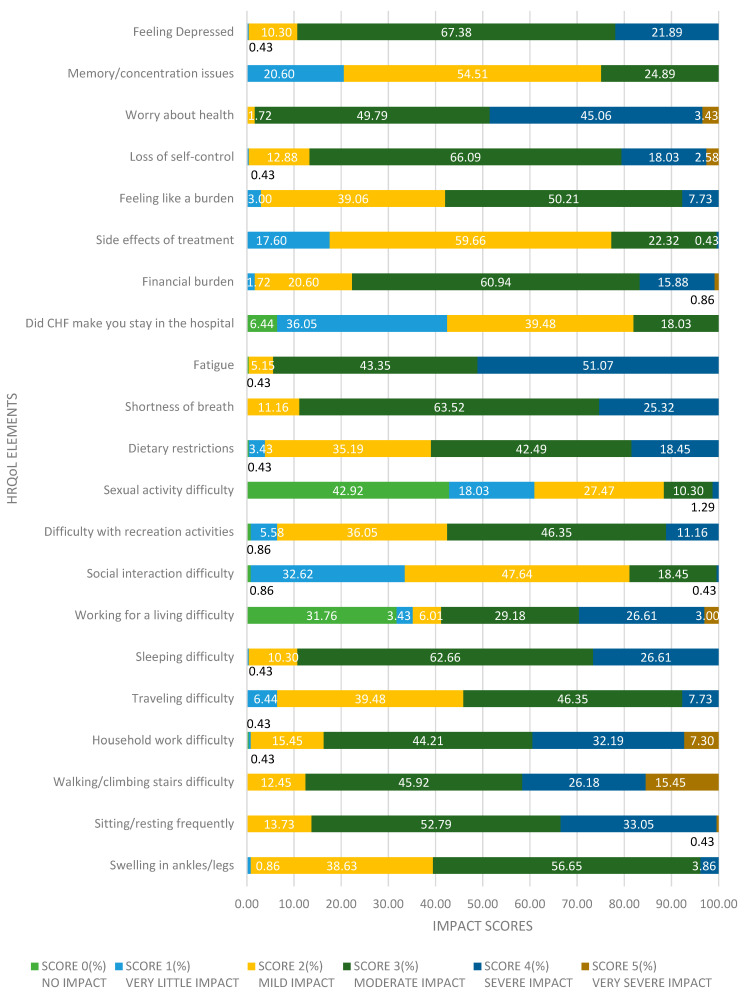
Overall distribution of MLHFQ score responses among CHF patients.

This section presents the item-wise distribution of responses as shown in Table 2 and Figure 1, to the 21 questions from the Minnesota Living with Heart Failure Questionnaire (MLHFQ), which captures how CHF impacts different aspects of a patient’s physical, emotional and social well-being. Each MLHFQ item is scored from 0 (no impact) to 5 (very significant impact). The responses are presented separately for patients with CHF with HFrEF and those with HFpEF, including both frequency and percentage distributions. These item-level insights provide a more detailed understanding of patient-reported symptoms, functional limitations and psychosocial burden in both subtypes of heart failure.

1. Physical Symptoms
  - Swelling in ankles/legs: Over half of the patients (56.7%, *n* = 132) reported a score of 3, indicating moderate swelling. An additional 38.6% (*n* = 90) reported a score of 2 (mild swelling). This suggests that peripheral edema is a prevalent issue but generally remains at a manageable level.
  - Sitting or resting frequently: A significant portion (52.8%, *n* = 123) experienced a moderate need to rest (score 3), and 33.0% (*n* = 77) reported a higher limitation (score 4). This reflects substantial fatigue or reduced stamina affecting daily functioning.
  - Walking/climbing stairs difficulty: Nearly half (45.9%, *n* = 107) reported moderate difficulty (score 3), while 26.2% (*n* = 61) and 15.5% (*n* = 36) experienced severe to very severe limitations (scores 4 and 5, respectively). This underscores mobility as a key challenge.
  - Fatigue: Among the most debilitating symptoms, fatigue was rated score 4 by 51.1% (*n* = 119) and score 3 by 43.3% (*n* = 101), indicating that a majority of patients experience moderate to severe exhaustion.
2. Sleep and Activity Limitations
  - Sleeping difficulty: Most participants (62.7%, *n* = 146) rated this as moderate (score 3), and 26.6% (*n* = 62) rated it as severe (score 4), reflecting disrupted sleep patterns common in CHF.
  - Household work difficulty: 44.2% (*n* = 103) reported score 3 and 32.2% (*n* = 75) reported score 4. This suggests limitations in fulfilling domestic responsibilities.
  - Travelling difficulty: 46.4% (*n* = 108) rated this moderately difficult (score 3), while 39.5% (*n* = 92) reported mild difficulty (score 2). Severe restrictions were less common (7.7%, score 4).
  - Recreational activities: 46.4% (*n* = 108) of patients experienced a moderate impact (score 3) and 36.1% (*n* = 84) had mild limitations (score 2), suggesting that leisure activities are notably affected but not severely curtailed for most.
  - Working for a living: Around 29.2% (*n* = 68) reported moderate difficulty (score 3), and 26.6% (*n* = 62) reported severe difficulty (score 4). Notably, 31.8% (*n* = 74) reported no impact (score 0), which may include retired or non-working individuals.
3. Emotional and Psychosocial Impact
  - Social interaction difficulty: 47.6% (*n* = 111) and 32.6% (*n* = 76) reported mild to moderate impact (scores 2 and 1, respectively), while only a small percentage indicated severe social limitations, suggesting CHF moderately interferes with interpersonal relations.
  - Feeling depressed: Depression was highly prevalent, with 67.4% (*n* = 157) scoring 3 and 21.9% (*n* = 51) scoring 4. This indicates a significant emotional burden affecting most patients.
  - Worry about health: Nearly all patients reported worry, with 49.8% (*n* = 116) scoring 3 and 45.1% (*n* = 105) scoring 4, highlighting high levels of health-related anxiety.
  - Feeling like a burden: 50.2% (*n* = 117) scored 3 and 39.1% (*n* = 91) scored 2, revealing that emotional guilt or perceived burden on caregivers is common.
  - Loss of self-control: A striking 66.1% (*n* = 154) reported a score of 3, and 18.0% (*n* = 42) scored 4, indicating that psychological feelings of helplessness are frequent and intense.
4. Cognitive and Economic Burden
  - Memory/concentration issues: Most patients (54.5%, *n* = 127) reported mild cognitive impact (score 2), with another 24.9% (*n* = 58) experiencing moderate difficulty (score 3), suggesting that cognitive symptoms are prevalent, though mostly not severe.
  - Financial burden: A large proportion of patients (60.9%, *n* = 142) rated financial impact at score 3 and 15.9% (*n* = 37) at score 4. This underlines the significant economic strain heart failure places on patients.
  - Hospital stays due to CHF: 39.5% (*n* = 92) reported a score of 3 and 36.1% (*n* = 84) a score of 2, reflecting frequent or impactful hospitalizations among this population.
  - Side effects of treatment: Most patients (59.7%, *n* = 139) reported mild treatment side effects (score 2), while 22.3% (*n* = 52) noted moderate ones (score 3), indicating treatment tolerability but with some burden.

The MLHFQ item-wise analysis reveals a complex picture of patient experience with CHF. Fatigue, breathlessness, sleeping difficulties and functional impairments like stair climbing and household work were among the most severely affected domains. Emotional and psychological aspects such as depression, health-related worry and feelings of loss of control were also highly prevalent. Cognitive and financial burdens further contributed to the overall reduction in health-related quality of life.

These findings emphasize the multidimensional impact of CHF on patients and highlight the need for holistic care approaches that address not only physical symptoms but also emotional, social and financial challenges.

**Figure 2:**
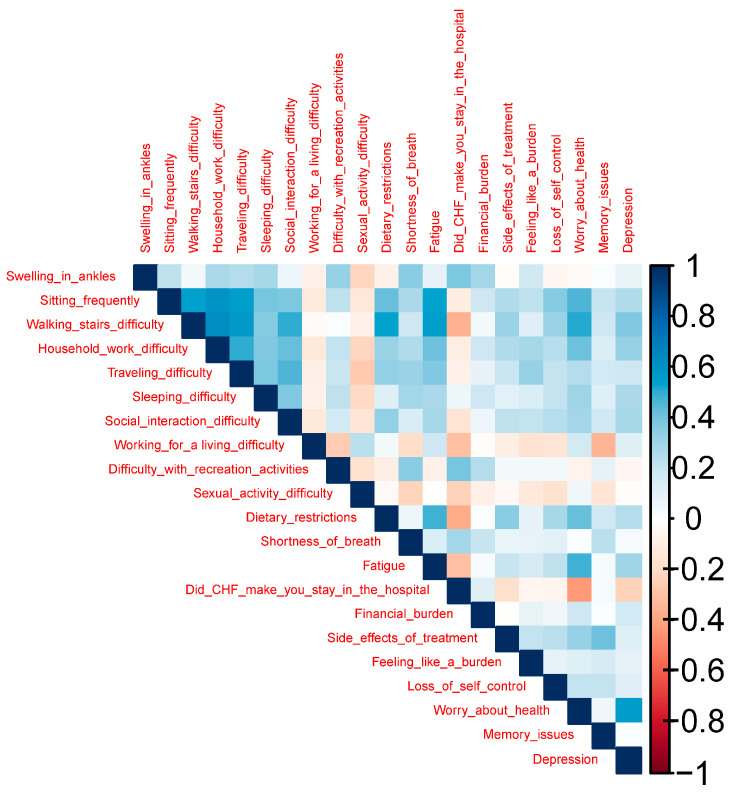
Correlation matrix heatmap of MLHFQ score.

The correlation matrix heatmap of MLHFQ Score is presented in Figure 2. Strong positive correlations are observed among functional difficulty variables such as walking stairs, household work, traveling, and sleeping, suggesting that challenges in one physical domain are often accompanied by difficulties in others. Similarly, emotional and psychological variables, namely depression, worry about health, feeling like a burden, and loss of self-control also cluster together, reflecting the interconnected nature of psychosocial distress. Depression, in particular, shows notable associations with memory issues and emotional burden, while fatigue demonstrates strong correlations with both physical and emotional domains, indicating its central role in overall HRQoL impairment. In contrast, variables like swelling in the ankles appear more isolated with weaker correlations, particularly with emotional or cognitive factors.

### 3.3. Descriptive Comparison of HRQoL Between HFrEF and HfpEF

HFpEF patients reported significantly higher HRQoL scores (mean = 61.4, SD = 3.94) than HFrEF patients (mean = 52.9, SD = 4.64). A score below 24 indicates a good quality of life, a score between 24 and 45 indicates a moderate quality of life, and a score above 45 indicates a poor quality of life [15]. The mean HRQoL score was 56.5 ± 6.05, reflecting a high symptom burden. Patients with HFpEF reported significantly poorer HRQoL (mean: 61.4 ± 3.94) compared to those with HFrEF (mean: 52.9 ± 4.64), with a large effect size (Cohen’s d = 1.95). A significant positive correlation was found between HRQoL score and age (r = 0.428; *p* < 0.001), indicating higher symptom burden among older adults. HRQoL also varied significantly across NYHA functional classes (χ^2^ = 69.9, *p* < 0.001, ε^2^ = 0.301, a large effect) and employment groups (χ^2^ = 17.0, *p* < 0.001, moderate effect).

### 3.4. Independent Samples t-Test for HRQoL

An independent samples *t*-test confirmed the HRQoL difference (t(231) = 14.7, *p* < 0.001, Cohen’s d = 1.95), showing a large effect size. Assumptions of normality through Shapiro–Wilk’s test (*p*-value > 0.05) and homogeneity through Levene’s test were met.

### 3.5. Factors Associated with HRQoL

**Figure 3:**
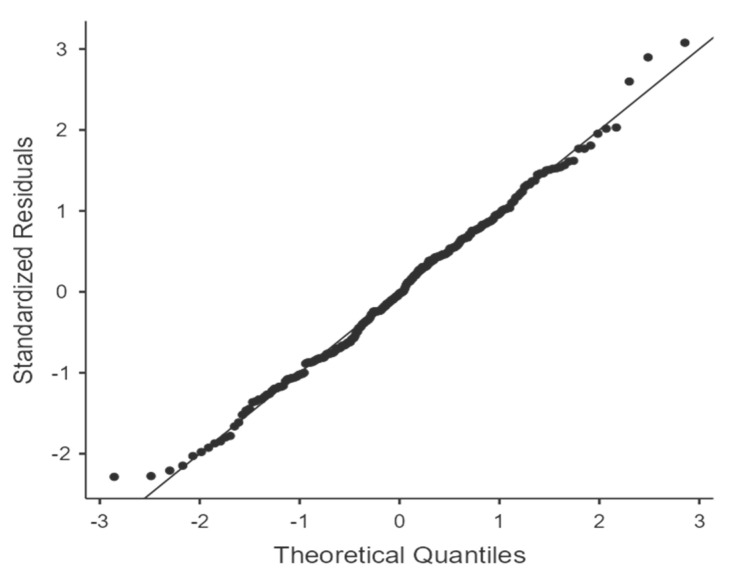
Q-Q plot.

Prior to conducting the multiple linear regression analysis, key assumptions were assessed to ensure the validity of the model. Normality of residuals was confirmed using three tests: the Shapiro–Wilk test (W = 0.994, *p* = 0.477), Kolmogorov–Smirnov test (*p* = 0.721), and Anderson–Darling test (*p* = 0.575), all indicating no significant deviation from normality. Homoscedasticity was evaluated through the Breusch–Pagan test (*p* = 0.907), suggesting constant variance of residuals. Although the Goldfeld–Quandt test returned a significant result (*p* = 0.018), this was not consistently supported by the Harrison–McCabe test (*p* = 0.130), and visual inspection of residual plots did not reveal substantial heteroskedasticity. Multicollinearity was assessed using variance inflation factors (VIFs), all of which were well below the threshold of concern (maximum VIF = 1.62), indicating no collinearity issues among predictors. Collectively, these diagnostics supported the appropriateness of the regression model. The Q-Q plot, as seen in Figure 3, indicates that the normality assumption of residuals is reasonably met. While there are minor deviations at the extremes, the overall distribution of residuals closely follows a normal distribution, supporting the use of parametric regression techniques.

Bivariate and multivariate (Table 3) analyses identified age, gender and ejection fraction type as significant predictors of HRQoL. Older patients and females reported higher HRQoL scores. Other sociodemographic variables (education, employment, marital status) were not significant in the regression model, although bivariate analysis indicated some associations. The adjusted R^2^ of the model is 0.323, along with the RMSE value of 4.86.

The stepwise multiple regression model (F(9,223) = 13.2, *p* < 0.001) explained 32.1% of the variance in HRQoL.

## 4. Discussion

### 4.1. Overall HRQoL Among CHF Patients

This study assessed the HRQoL in patients with CHF, focusing on physical and emotional dimensions in individuals with HFpEF and HFrEF. The overall mean HRQoL score was 56.5 ± 6.05, indicating a moderate symptom burden. These findings are comparable to an Indian study reporting a mean score of 53 ± 3.15 using the MLHFQ scale [16]. Similarly, research in Punjab documented moderate impairment across both domains, highlighting daily limitations faced by CHF patients [17].

### 4.2. HRQoL Differences Between HFpEF and HFrEF

A key finding was that HFpEF patients reported significantly poorer HRQoL than those with HFrEF, with a large effect size. This aligns with prior research in India showing a greater quality-of-life burden in HFpEF patients [18]. Despite having preserved systolic function, HFpEF patients frequently report worse symptoms and limitations, likely due to older age, higher comorbidity rates, and psychosocial stressors [12,19]. These factors appear to contribute more to quality-of-life outcomes than ejection fraction alone. These results emphasize the need to incorporate routine HRQoL assessments into standard CHF care, especially in hospital and cardiology settings. Identifying patients with higher symptom burden—particularly older adults, females, and those with HFpEF—can enable clinicians to deliver personalized interventions that address not only clinical management but also psychological and social support.

Although patients with HFpEF exhibit preserved systolic function, several physiological and psychosocial factors may contribute to the significantly poorer HRQoL observed in this group. One key contributor is diastolic dysfunction, which is the hallmark of HFpEF. Unlike HFrEF, where pump failure predominates, HFpEF is characterized by impaired ventricular relaxation and increased filling pressures, leading to exercise intolerance, pulmonary congestion, and exertional dyspnea—symptoms that severely affect daily functioning and well-being. Moreover, chronotropic incompetence and vascular stiffness, both common in HFpEF, can further limit functional capacity and exacerbate fatigue and breathlessness.

HFpEF patients are typically older adults, and age itself is strongly associated with diminished physiological reserve and increased frailty—a state of vulnerability to stressors that compounds the impact of CHF symptoms. Frailty in HFpEF has been associated with increased risk of hospitalization, disability, and poor quality of life independent of ejection fraction. Additionally, the HFpEF phenotype is often accompanied by multimorbidity, including hypertension, obesity, diabetes, chronic kidney disease, and atrial fibrillation. These coexisting conditions impose complex self-management demands and synergistically worsen both physical and emotional aspects of HRQoL.

Psychosocially, older HFpEF patients may also face greater social isolation, reduced mobility, and financial dependence, which exacerbate feelings of depression, helplessness, and being a burden—all of which were prominently reported in our item-wise MLHFQ analysis. In contrast, some HFrEF patients—though at higher risk of mortality—may receive more aggressive guideline-directed therapy and advanced cardiac interventions, potentially leading to improved symptom control and QoL.

Therefore, despite the term “preserved ejection fraction,” the lived experience of HFpEF patients often involves a higher burden of unaddressed symptoms and psychosocial distress, which may not be captured through traditional clinical metrics alone. These insights underscore the importance of adopting comprehensive, multidisciplinary, and age-sensitive care strategies for HFpEF patients, with routine HRQoL assessments to guide individualized care.

### 4.3. Sociodemographic Correlates of HRQoL

Several sociodemographic variables were significantly associated with HRQoL. Advancing age was negatively correlated with HRQoL, aligning with similar results of reduced functioning observed in older individuals [18]. This may be linked not only to biological ageing but also to comorbidities, diminished resilience and social isolation. Supporting this, a study in Ethiopia identified age as a key predictor of reduced HRQoL [20].

Gender differences were evident, with female patients experiencing significantly lower HRQoL. This trend has been well-documented, with studies noting that women with CHF tend to report greater physical limitations and emotional distress [18,21,22]. These findings underscore the importance of gender-sensitive care strategies, especially those addressing psychological well-being in female patients.

Educational attainment emerged as another influential factor. Individuals with lower education levels exhibited poorer HRQoL, a trend supported by studies highlighting limited health literacy among less-educated CHF patients [17,18]. It was found that lower education negatively impacts self-care behaviors and overall HRQoL [23]. Improving health education and literacy may enhance disease management and outcomes in this group.

Employment status also played a significant role. Unemployed patients had lower HRQoL scores, reflecting the psychological and socioeconomic burden of joblessness. Although limited Indian data explore this link, studies have noted high unemployment rates among CHF patients [17,18]. Internationally, unemployment has been associated with worse symptom severity and emotional well-being, suggesting a need for vocational and social support interventions [2].

Marital status was another relevant factor. Unmarried or widowed individuals reported poorer HRQoL, potentially due to the absence of emotional and social support. Although one Indian study did not find a significant association, international evidence supports the idea that social connections buffer against stress and improve chronic disease outcomes [10,17]. Tailored support for socially isolated patients could therefore help mitigate some of the HRQoL decline.

NYHA functional class was a strong clinical predictor of HRQoL. Patients in advanced classes (III and IV) experienced significantly worse outcomes in both physical and emotional domains. This is consistent with previous findings where a higher functional class correlated with more severe limitations and emotional burden [16,20]. The progression of heart failure symptoms often leads to reduced independence and heightened psychological distress, thereby diminishing overall quality of life.

This study highlights the multidimensional impact of CHF, illustrating the importance of holistic and patient-centric care approaches. Beyond pharmacological interventions, management should incorporate psychosocial support, education and structured rehabilitation. Particular attention should be directed towards high-risk groups such as elderly patients, females, individuals with lower education, unemployed persons and those in NYHA class III or IV to optimize outcomes.

While several studies, both globally and within India, have examined HRQoL among CHF patients with HFpEF and HFrEF, this study makes distinct contributions. Firstly, it is one of the first to conduct a detailed, item-wise analysis of the Minnesota Living with Heart Failure Questionnaire (MLHFQ) responses specifically from a quaternary care setting in coastal Karnataka. This offers granular insights into patient experiences across physical, emotional, and socioeconomic domains. Secondly, while prior studies have reported associations of HRQoL with demographic variables, this study contextualizes those findings by linking them to real-world challenges such as employment status, educational attainment, and perceived caregiver burden in an LMIC setting. These factors, often underexplored, reflect how structural determinants intersect with disease subtype to shape lived experience. Lastly, the study’s focus on stratified care implications—especially for older adults and women—helps build evidence for locally-relevant, equity-oriented CHF care strategies, addressing a critical gap in the literature.

## 5. Limitations

A key limitation of this study lies in the adoption of a convenience sampling approach, which may have introduced selection bias and compromised the external validity of the findings. The absence of random selection limits the extent to which the results may be extrapolated to the general CHF population, particularly to individuals who do not access care at the study site or who may differ in important demographic or clinical characteristics. Another limitation of this study is its single-center design, which may limit the generalizability of the findings. As all participants were recruited from a single clinical site, the sample may disproportionately reflect the characteristics of patients who seek care in that specific setting, potentially overrepresenting individuals with more advanced or symptomatic disease.

A further limitation is the limited range of variables included in the stepwise multivariable regression model. Key clinical factors such as comorbidities (e.g., diabetes, CKD, hypertension), medication use, and more detailed measures of heart failure severity were not included due to inconsistent data availability. This may have resulted in residual confounding, affecting the accuracy of the identified predictors. Future studies should aim to incorporate these variables for more robust analyses.

## 6. Conclusions

In conclusion, this study highlights that CHF patients experience moderate to high symptom burdens impacting their physical and emotional well-being. Patients with HFpEF exhibited poorer HRQoL compared to those with HFrEF. Older age, female gender, lower education, unemployment, marital status and higher NYHA functional class were significantly associated with poorer HRQoL. These findings suggest the urgent need for integrated, patient-centered management strategies aimed at improving the quality of life among CHF patients.

## Figures and Tables

**Table 1:** Sociodemographic and clinical characteristics between HFrEF and HFpEF patients.

Characteristics		HFrEF		HFpEF
	Frequency(n)	Percentage (%)	Frequency(n)	Percentage (%)
**Ejection fraction type**	136	58.4	97	41.6
**Age (years old)**				
25–35	2	0.9	0	0
36–45	11	4.7	0	0
46–55	36	15.5	24	10.3
56–65	50	21.5	44	18.9
66–75	29	12.4	25	10.7
76–85	8	3.4	4	1.7
**Gender**				
Male	121	51.9	44	18.9
Female	15	6.4	53	22.7
**Employment Status**				
Unemployed	39	16.7	29	12.4
Employed	61	26.2	40	17.2
Retired	36	15.5	28	12
**Education Level**				
No Formal Education	13	5.6	10	4.3
Primary school	55	23.6	41	17.6
High School	46	19.7	36	15.5
Higher Education (Diploma/University)	22	9.4	10	4.3
**NYHA functional class**				
NYHA I	0	0	0	0
NYHA II	41	17.6	26	11.2
NYHA III	73	31.3	64	27.5
NYHA IV	22	9.4	7	3
**Duration of Congestive HF disease (Years)**				
1	13	5.6	15	6.4
2	41	17.6	34	14.6
3	36	15.5	27	11.6
4	25	10.7	13	5.6
5	16	6.9	8	3.4
6	5	2.1	0	0
**Marital Status**				
Unmarried	3	1.3	0	0
Married	123	52.8	80	34.3
Widowed	10	4.3	17	7.3

**Table 2:** Key MLHFQ item differences between HFrEF and HFpEF.

MLHFQ Item	HFrEF Mean Score	HFpEF Mean Score	Difference
Fatigue	4.1	2.1	2.0
Shortness of Breath	3.9	2.6	1.3
Depression	3.6	2.7	0.9
Travel Difficulty	3.3	2.4	0.9

**Table 3:** Multiple regression coefficients for predicting HRQoL.

			95% Confidence Interval			
Predictor	Estimate	SE	Lower	Upper	t	*p*
Intercept	41.203	3.1182	35.058	47.348	13.214	<0.001
Age	0.311	0.0565	0.2	0.422	5.505	<0.001
Gender:						
Male—Female	−4.591	0.7803	−6.128	−3.053	−5.883	<0.001
Educational Level:						
Higher Education(Diploma/University) High School	0.43	1.1348	−1.806	2.667	0.379	0.705
No Formal Education—High School	2.332	1.369	−0.366	5.03	1.703	1.090
Primary school—High School	0.739	0.7958	−0.829	2.307	1929	0.354
Employment Status:						
Retired—Employed	−0.703	1.0308	−2.735	1.328	−0.682	0.496
Unemployed—Employed	−0.688	0.909	−2.479	1.103	−0.757	0.450
Marital Status:						
Unmarried—Married	−2.049	3.1519	−8.26	4.162	−0.65	0.516
Widowed—Married	−1.445	1.1746	−3.759	0.87	−1.23	0.22
Ejection Fraction Type						
HFrEF-HFpEF	−7.902	0.5752	−9.035	−6.768	−13.739	<0.001

## Data Availability

The original contributions presented in this study are included in the article/Supplementary Materials. Further inquiries can be directed to the corresponding author(s).

## Supplementary Materials

The following supporting information can be downloaded at: https://www.mdpi.com/article/10.3390/healthcare13151874/s1, HRQOL_segregated data.
